# Long-term humoral response following Delta and Omicron BA.1 co-infection

**DOI:** 10.1038/s41541-023-00652-8

**Published:** 2023-04-20

**Authors:** Carla Saade, Bruno Pozzetto, Melyssa Yaugel-Novoa, Guy Oriol, Laurence Josset, Bruno Lina, Stéphane Paul, Antonin Bal, Sophie Trouillet-Assant

**Affiliations:** 1 CIRI—Centre International de Recherche en Infectiologie, Université Claude Bernard Lyon 1, Inserm, U1111, CNRS, UMR5308, ENS Lyon, Université Jean Monnet de Saint-Etienne, Lyon, 69007 France; 2 Immunology Laboratory, CIC1408, CHU Saint-Etienne, Saint-Etienne, 42055 France; 3 Joint Research Unit Civils Hospices of Lyon-bioMérieux, Hospices Civils de Lyon, Hôpital Lyon Sud, Pierre-Bénite, 69310 France; 4 Laboratoire de Virologie, Institut des Agents Infectieux, Centre National de Référence des virus des infections respiratoires, Hospices Civils de Lyon, F-69004 Lyon, France; 5 GenEPII Sequencing Platform, Institut des Agents Infectieux, Hospices Civils de Lyon, F-69004 Lyon, France

**Keywords:** Viral infection, Infection

## Abstract

This study reports the 6-month humoral immune response in vaccinated patients concomitantly infected with Delta and Omicron BA.1 variants of SARS-CoV-2. Interestingly, the simultaneous exposure to the Delta and BA.1 S proteins does not confer an additional immune advantage compared to exposure to the BA.1 S protein alone.

Bivalent vaccines containing two different sequences of the Spike (S) protein have been recently approved. First studies evaluating the short-term immune response after the administration of bivalent vaccines have reported contradictory results^1–5^. Moreover, the long-term immunity outcomes of this bivalent vaccine need further investigation, especially given the waning of the humoral response. Others and we have reported cases of simultaneous infection by the Delta and BA.1 variants, both having caused major COVID-19 epidemic waves worldwide^6,7^. The present report describes the long-term humoral response of patients infected at the same time with the Delta and BA.1 variants. The main goal of the study was to compare breakthrough infections with only one or two variants simultaneously.

We included two groups of vaccinated individuals with a breakthrough infection (BA.1 in the first group [*n* = 9] and Delta and BA.1 co-infection in the second group [*n* = 9, Supplementary Flow Chart]). Co-infection refers to the simultaneous detection of genomes belonging to two different SARS-CoV-2 variants as determined by sequencing^6^. All infected individuals experienced a mild form of COVID-19. We included 9 COVID-19-naïve individuals who received three doses of the BNT162b2 vaccine as a control group (Supplementary Table 1 for demographic data and Supplementary Table 2 for individual immunization/infection history). All individuals received the BNT162b2 vaccine to minimize differences between vaccination schemes. Blood sampling for humoral immunity investigation was performed 6 months after breakthrough infection for infected individuals or 6 months after the third vaccine injection for COVID-19-naïve individuals to take into account waning of the humoral immune response (Supplementary Table 1). Details regarding the interval between last immunization (infection for individuals with a breakthrough infection and vaccination for fully vaccinated individuals) and blood sampling for each participant, as well as immunization scheme are provided in Supplementary Table 2.

There was no significant difference in anti-receptor binding domain (RBD) IgG and anti-S1 IgA levels among individuals with a breakthrough infection caused by Delta and BA.1 or by BA.1 only; however, these levels were 4.13 and 10.83 -fold lower, respectively, among COVID-19-naïve individuals than in those with hybrid immunity (Fig. 1a, b).

**Fig. 1:**
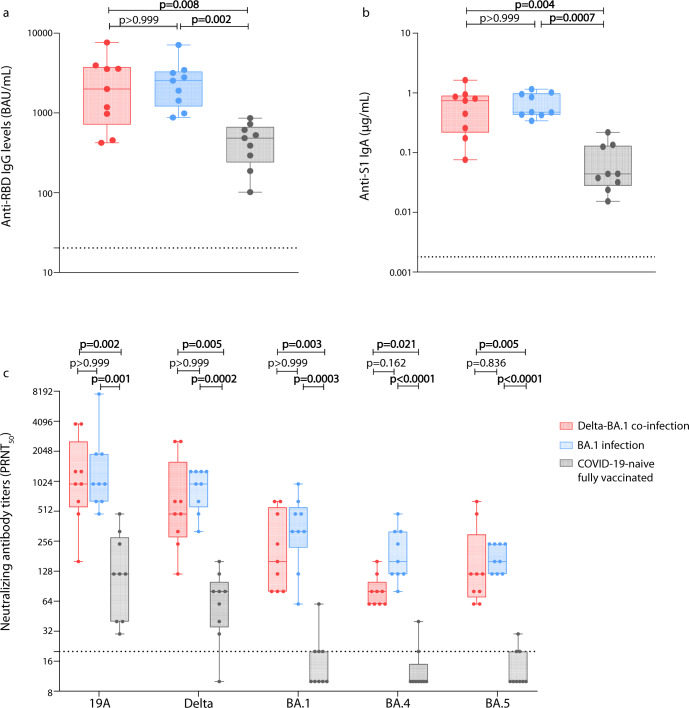
Humoral immune response 6 months after last immunization in individuals vaccinated and then co-infected with Delta and BA.1, or vaccinated and then infected with BA.1, or in COVID-19-naïve fully-vaccinated individuals. Blood samples were obtained 6 months after infection for the first two groups or 6 months after the third injection for the third group. Anti-RBD IgG levels were measured using the commercially available bioMérieux Vidas SARS-CoV-2 IgG diagnosis kit according to manufacturer’s recommendations and expressed in binding antibody unit (BAU)/mL. The dotted line represents the threshold of positivity (≥ 20.33 BAU/mL; **a**). Anti-S1 IgA levels were measured using an ELISA test and expressed in µg/mL. The dotted line represents the positivity threshold (≥0.0018 µg/mL; **b**). Neutralizing antibody titers against live SARS-CoV-2 isolates using a 50% plaque reduction neutralization test (PRNT_50_). The isolates used for these experiments were 19A, Delta, Omicron BA.1, BA.4, and BA.5; their GISAID accession numbers are EPI_ISL_1707038, EPI_ISL_1904989, EPI_ISL_7608613, EPI_ISL_12396843, and EPI_ISL_12852091, respectively. The dotted line represents the positivity threshold (titer ≥ 20; **c**). Data are represented as box and whiskers plot; in each plot, the dots indicate individual samples, the upper and lower limits of the box plot represent the interquartile range [IQR] and the middle line represents the median. Whiskers represent the maximum and minimum value in each plot. All box and whiskers plots represent *n* = 9 biologically independent samples. A Kruskal–Wallis test followed by Dunn’s multiple comparison tests were performed to assess differences between the three groups.

Neutralizing antibody titers were significantly lower among COVID-19-naïve individuals for all viral variants compared to BA.1-infected and Delta-BA.1-co-infected individuals. Compared to COVID-19-naïve individuals, the median neutralizing antibody titer of BA.1-infected individuals was 8-fold higher for the 19A isolate and 12-fold higher for the BA.5 isolate, and it was 8-fold higher for the 19A isolate and 16-fold higher for the BA.5 isolate in Delta-BA.1 co-infected individuals. Interestingly, no significant difference was observed between the two groups of individuals with a breakthrough infection (Fig. 1c).

In addition, we calculated the geometric mean titer (GMT) ratio between the three groups for all the tested parameters (Supplementary Table 3). These ratios were similar between the Delta-BA.1 co-infected and BA.1 infected individuals, but a marked difference was seen when compared to the COVID-19-naïve fully vaccinated individuals (ratios ranged from 4 to 20). Moreover, a multiple linear regression analysis was performed with two adjustment variables, i.e., interval between last immunization and blood sampling for humoral investigation, and age at blood sampling. This analysis found that these variables did not influence the humoral response (Supplementary Data).

Results reported herein show an enhanced humoral response after breakthrough infections caused by one or two variants. These results confirm the advantage conferred by so-called hybrid immunity that has already been shown to induce a more potent long-term humoral immune response against SARS-CoV-2 in comparison to that induced by vaccination only^8^. In addition, the absence of significant difference in anti-S total antibodies and neutralization capacity between individuals with a single or a dual breakthrough infection would suggest that a simultaneous exposure to the Delta and Omicron BA.1 variants does not confer an additional immune advantage in terms of humoral immunity.

The present study does, however, have certain limitations. The most evident is the small size of the effective in each group due to the rarity of simultaneous infection with two different SARS-CoV-2 variants; another limitation is that comorbidities were more frequent in the COVID-19-naïve vaccinated group, even if these exhibited only one comorbidity each (Supplementary Table 1).

In conclusion, the results reported herein suggest the possibility of an immune imprinting in the context of anti-SARS-CoV-2 humoral response, also known as original antigenic sin. This phenomenon is in reference to a limited immune response against a new antigen variant after an exposure to the initial one^2,3^. However, extrapolations of these findings to bivalent vaccines should be made with caution since a spontaneous infection is not equivalent to vaccination in terms of route of exposure as well as antigen load and diversity. It is also of note that contradictory results have been reported in terms of the additional protection provided by these new vaccines^2–5^; long-term studies using bivalent vaccines, which are now the standard of care, will give better insight into their effectiveness.

## Methods

### Study design and ethics

We included two groups of vaccinated individuals with a breakthrough infection (BA.1 in the first group [*n* = 9] and Delta and BA.1 co-infection in the second group [*n* = 9, Supplementary Flow Chart]). We included 9 COVID-19-naïve individuals who received three doses of the BNT162b2 vaccine as a control group. Blood sampling for humoral immunity investigation was performed 6 months after breakthrough infection for infected individuals or 6 months after the third vaccine injection for COVID-19-naïve individuals. Participants were included from two clinical studies registered on ClinicalTrials.gov (NCT05060939, NCT04341142). Written informed consent was obtained from all participants and approval was obtained from the regional review board in April 2020 (*Comité de Protection des Personnes Sud Méditerranée I*, Marseille, France; ID-RCB 2020-A00932-37; ID-RCB 2021-A01877-34).

### Laboratory methods

VIDAS® SARS-COV-2 IgG II (9COG, ref 424114) is an automated semi‑quantitative assay for use on the VIDAS® family of instruments, for the detection of immunoglobulin G (IgG) specific for the SARS‑CoV‑2 receptor-binding domain (RBD) of the spike protein in human serum or plasma (lithium heparin) using the Enzyme Linked Fluorescent Assay (ELFA) technique. The VIDAS® SARS-COV-2 IgG II (9COG) is a commercialized validated assay with a CE marking. The test was carried out according to the protocol recommended by the manufacturer and expressed in binding antibody units (BAU)/mL.

Regarding the IgA ELISA assay, the test was validated with negative and positive samples as well as a calibration range with a recombinant IgA. In brief, high binding 96–half-well plates (#2310 M; NUNC) were coated with 100 μL per well of a spike protein solution (1 μg/mL; #40591-V08H Spike S1-RBD Sino Biologicals) in PBS overnight at 4 °C. Plates were washed with washing buffer containing 1X PBS with 0.05% Tween 20 (Sigma-Aldrich) and incubated with 170 μL of blocking buffer per well containing 1X PBS with 3% fat milk powder and 0.05% Tween 20 (Sigma-Aldrich) for 1 h at room temperature. Immediately after blocking, recombinant anti-RBD IgA (B Cell Design #IB3C4 PV) or serum samples diluted in PBS were added and incubated for 1 h at 37 °C. Plasma samples were assayed at a 1:100 starting dilution and seven additional threefold serial dilutions. Recombinant human anti-RBD IgA was used to perform a calibration curve starting at 1.5 µg/mL. Plates were washed and then incubated with anti-human IgA (A0295; Sigma-Aldrich) secondary Ab conjugated to horseradish peroxidase (HRP) in blocking buffer at 1:10000. Plates were developed by addition of the HRP substrate, 3,3′,5,5″-tetramethylbenzidine (TMB; 34021; Thermo Fisher Scientific), for 10 min, and then the developing reaction was stopped by adding 50 μl of 1 M HCl. Optical density units were measured at 450 nm in a microplate reader (TECAN). For serum samples, a positive control (serum pool from critical COVID patients, diluted 200-fold in PBS) and a negative control (pool of historical serum samples) were added in duplicate to each run. After deduction of the background, a relative content in IgA Equivalent (ng/ml Eq) was calculated using the calibration curve. The limit of detection of the assay was 0.1 ng/mL Eq. All serum samples were tested as duplicates.

A live virus neutralization test measuring neutralizing antibodies titers against 19A, Delta and Omicron BA.1, BA.4, and BA.5 isolates and their GISAID accession numbers are EPI_ISL_1707038, EPI_ISL_1904989, EPI_ISL_7608613, EPI_ISL_12396843, and EPI_ISL_12852091 respectively. Viral variants used for these experiments were cultured on Vero-E6 cells. Each serum tested was diluted 1:10 and serial twofold dilutions were mixed with an equal volume (100 µL) of virus. After gentle shaking and an incubation for 30 min at room temperature, 150 µL of each mixture was transferred to 96-well microplates covered with Vero-E6 cells. Then, the plates were incubated at 37 °C in an atmosphere containing 5% CO_2_. Measurements were obtained microscopically 5–6 days later when the cytopathic effect of the virus control reached ~100 50% tissue culture infectious dose (TCID_50_)/150 µL. The serum was considered to have protected the cells if >50% of the cell layer was preserved. The neutralizing titer was expressed as the inverse of the higher serum dilution that protected the cells. All serum samples were tested as duplicates.

### Statistical analyses

To assess the differences in anti-RBD IgG levels, anti-S1 IgA and neutralizing antibody titers between the three groups, a Kruskal–Wallis test followed by Dunn’s multiple comparison tests were performed. A multiple linear regression was performed to assess the impact of two adjustment variables, (i) delay between last immunization and blood sampling and (ii) age at blood sampling, on the humoral response described. Statistical analyses were performed using the GraphPad Prism® software (version 8; GraphPad software, La Jolla, CA, USA) and R software, version 3.6.1 (R Foundation for Statistical Computing, Vienna, Austria).

### Reporting summary

Further information on research design is available in the Nature Research Reporting Summary linked to this article.

## Supplementary information

Supplementary material

REPORTING SUMMARY

## Data Availability

The datasets generated and/or analyzed during the current study are available from the corresponding author on reasonable request. GISAID accession numbers for the 19A, Delta, BA.1, BA.4, and BA.5 strains used are EPI_ISL_1707038, EPI_ISL_1904989, EPI_ISL_7608613, EPI_ISL_12396843, and EPI_ISL_12852091, respectively.
