# Supplementary material for: Long-term humoral response following Delta and Omicron BA.1 co-infection
Source: NPJ Vaccines. 2023 Apr 20;8:57. doi: 10.1038/s41541-023-00652-8 (PMC10116110; doi:10.1038/s41541-023-00652-8)
Supplement: Supplementary file 1 — Supplementary material [file 41541_2023_652_MOESM1_ESM.pdf]

Supplementary material

Supplementary Table 1: Demographic and clinical characteristics of the 27 healthcare workers included in the study.

	Delta-BA.1 co-infection (n=9)	BA.1 Infection (n=9)	COVID-19-naïve individuals (n=9)
Female, n (%)	3 (33)	6 (66)	6 (66)
Age at blood sampling, years, median [IQR]	32 [23-40]	49 [29-54]	51 [48-64]
Interval between last immunization[1] and blood sampling for humoral immunity investigation, days, median [IQR]	203 [197-212]	193 [208-166]	179 [178-183]
Comorbidities, n (%)	1 (11)	1 (11)	6 (66)
- hypertension	0	0	1 (11)
- diabetes	0	0	1 (11)
- hypothyroidism	0	0	1 (11)
- vascular disease	0	1 (11)	0
- chronic lung disease	1 (11)	0	2 (22)
- heterozygous sickle cell disease	0	0	1 (11)
Vaccination scheme, n (%)			
- 3 injections of BNT162b2[2]	2 (22)	3 (33)	9 (100)
- 2 injections of BNT162b2	6 (66)	6 (66)	0
- 1 injection of BNT162b2	1 (11)	0	0
Previous infection, n (%)	2 (22)	2 (22)	0

IQR: interquartile range.
[1] Last immunization corresponds to the infection for both groups with a breakthrough infection and the third vaccine injection for the COVID-19-naïve fully vaccinated group.
[2] BNT162b2 corresponds to the Pfizer-BioNTech monovalent SARS-CoV-2 vaccine.

Supplementary Table 2. Individual characteristics of the 27 healthcare workers included into the study.

Subject	Last immunization[1]	Age at blood sampling, years	Sex	Immunization scheme	Date of first vaccine injection	Date of second vaccine injection	Date of third vaccine injection	Date of previous infection	Date of breakthrough infection diagnosis	Date of blood sampling for humoral investigation	Delay last immunization[1]-blood sampling for humoral investigation, days
**Delta-BA.1 co-infection (n=9)**											
Subject-1	Simultaneous Delta and BA.1 breakthrough infection	26	Male	2 injections of BNT162b2	07/27/2021	08/18/2021	-	-	12/20/2021	07/23/2022	215
Subject-2	Simultaneous Delta and BA.1 breakthrough infection	20	Male	2 injections of BNT162b2	06/2021	07/28/2021	-	-	12/27/2021	07/22/2022	207
Subject-3	Simultaneous Delta and BA.1 breakthrough infection	43	Female	3 injections of BNT162b2	03/2021	04/08/2021	12/01/2021	-	12/27/2021	07/25/2022	210
Subject-4	Simultaneous Delta and BA.1 breakthrough infection	49	Male	3 injections of BNT162b2	06/2021	07/12/2021	12/12/2021	-	12/27/2021	08/01/2022	217
Subject-5	Simultaneous Delta and BA.1 breakthrough infection	38	Male	2 injections of BNT162b2	08/18/2021	09/2021	-	-	12/31/2021	07/21/2022	202
Subject-6	Simultaneous Delta and BA.1 breakthrough infection	18	Female	2 injections of BNT162b2	09/2021	10/07/2021	-	-	01/03/2022	07/25/2022	203
Subject-7	Simultaneous Delta and BA.1 breakthrough infection	26	Female	2 injections of BNT162b2 and previous breakthrough infection	06/2021	07/2021	-	12/16/2021	01/04/2022	07/21/2022	198
Subject-8	Simultaneous Delta and BA.1 breakthrough infection	35	Male	2 injections of BNT162b2	07/2021	07/22/2021	-	-	01/17/2022	07/23/2022	187
Subject-9	Simultaneous Delta and BA.1 breakthrough infection	32	Male	Previous infection followed by 1 injection of BNT162b2	07/07/2021	-	-	11/2020	01/18/2022	08/03/2022	197
**BA.1 infection (n=9)**											
Subject-1	BA.1 breakthrough infection	58	Male	3 injections of BNT162b2	09/01/2020	02/01/2021	10/14/2021	-	01/16/2022	06/21/2022	156
Subject-2	BA.1 breakthrough infection	26	Male	2 injections of BNT162b2	01/16/2021	02/25/2021	-	-	12/18/2021	07/21/2022	215
Subject-3	BA.1 breakthrough infection	33	Male	2 injections of BNT162b2	12/01/2020	02/08/2021	-	-	12/19/2021	06/30/2022	193
Subject-4	BA.1 breakthrough infection	49	Female	2 injections of BNT162b2 and previous breakthrough infection	06/01/2021	07/01/2021	-	10/01/2021	01/03/2022	06/23/2022	171
Subject-5	BA.1 breakthrough infection	25	Female	3 injections of BNT162b2	01/22/2021	02/22/2021	11/24/2021	-	01/24/2022	08/04/2022	192
Subject-6	BA.1 breakthrough infection	54	Female	3 injections of BNT162b2	06/01/2021	07/01/2021	10/01/2021	-	01/18/2022	08/22/2022	216
Subject-7	BA.1 breakthrough infection	54	Female	2 injections of BNT162b2	09/15/2021	10/15/2021	-	-	01/16/2022	07/28/2022	193
Subject-8	BA.1 breakthrough infection	51	Female	2 injections of BNT162b2	07/16/2021	08/17/2021	-	-	01/16/2022	08/05/2022	201

Subject-9	BA.1 breakthrough infection	35	Female	Previous infection followed by 2 injections of BNT162b2	04/01/2021	11/01/2021	-	03/01/2020	01/23/2022	07/04/2022	162
**COVID-19-naïve individuals (n=9)**											
Subject-1	Third injection of BNT162b2	49	Female	3 injections of BNT162b2	01/20/2021	02/26/2021	10/29/2021	-	-	04/26/2022	179
Subject-2	Third injection of BNT162b2	51	Female	3 injections of BNT162b2	01/20/2021	02/22/2021	11/29/2021	-	-	05/30/2022	182
Subject-3	Third injection of BNT162b2	77	Male	3 injections of BNT162b2	01/20/2021	02/17/2021	11/25/2021	-	-	05/23/2022	179
Subject-4	Third injection of BNT162b2	67	Female	3 injections of BNT162b2	01/21/2021	02/18/2021	09/14/2021	-	-	03/21/2022	188
Subject-5	Third injection of BNT162b2	58	Female	3 injections of BNT162b2	01/21/2021	02/19/2021	11/19/2021	-	-	05/17/2022	179
Subject-6	Third injection of BNT162b2	62	Female	3 injections of BNT162b2	01/22/2021	02/26/2021	11/19/2021	-	-	05/16/2022	178
Subject-7	Third injection of BNT162b2	50	Male	3 injections of BNT162b2	01/25/2021	02/22/2021	11/19/2021	-	-	05/12/2022	174
Subject-8	Third injection of BNT162b2	48	Female	3 injections of BNT162b2	02/01/2021	02/22/2021	10/22/2021	-	-	04/21/2022	181
Subject-9	Third injection of BNT162b2	38	Male	3 injections of BNT162b2	01/26/2021	02/16/2021	11/27/2021	-	-	05/31/2022	185

[1] Last immunization corresponds to the infection for both groups with a breakthrough infection and the third vaccine injection for the COVID-19-naïve fully vaccinated group.

Supplementary Table 3. Results of the serological tests in the three groups of participants.

Group	Test	GMT[1] (95% CI)[2]	GMT ratio		
			1 *vs* 2	1 *vs* 3	2 *vs* 3
	**Anti-RBD IgG (BAU/mL)**				
**1**	Delta-BA.1 co-infected	1771 (813-3855)	0.80	4.53	5.68
**2**	BA.1 infected	2220 (1336-3689)			
**3**	COVID-19-naive fully vaccinated	391 (230-664)			
	**Anti-S1 IgA (µg/mL)**				
**1**	Delta-BA.1 co-infected	0.47 (0.22-1.00)	0.77	9.00	11.76
**2**	BA.1 infected	0.62 (0.44-0.88)			
**3**	COVID-19-naive fully vaccinated	0.05 (0.03-0.10)			
	**Neutralizing antibodies (titer)**				
	**19A**				
**1**	Delta-BA.1 co-infected	1010 (472-2162)	0.85	8.92	10.55
**2**	BA.1 infected	1194 (625-2280)			
**3**	COVID-19-naive fully vaccinated	113 (54-240)			
	**Delta**				
**1**	Delta-BA.1 co-infected	563 (260-1221)	0.66	9.76	14.81
**2**	BA.1 infected	855 (579-1262)			
**3**	COVID-19-naive fully vaccinated	58 (30-109)			
	**BA.1**				
**1**	Delta-BA.1 co-infected	198 (100-392)	0.62	12.87	20.70
**2**	BA.1 infected	318 (166-612)			
**3**	COVID-19-naive fully vaccinated	15 (10-25)			
	**BA.4**				
**1**	Delta-BA.1 co-infected	80 (61-104)	0.43	6.31	14.73
**2**	BA.1 infected	186 (117-294)			
**3**	COVID-19-naive fully vaccinated	13 (9-18)			
	**BA.5**				
**1**	Delta-BA.1 co-infected	132 (68-256)	0.76	10.02	13.21
**2**	BA.1 infected	174 (136-223)			
**3**	COVID-19-naive fully vaccinated	13 (9-18)			

[1] GMT: Geometric mean titer

[2] 95% CI: 95% confidence interval

NB: GMT results were rounded to the unit for the first and the third parameters tested and to two digits after unit for the second one. GMT ratios were rounded to two digits after the unit for all parameters.

Supplementary Data:

Since demographical parameters were not similar between the three groups we performed a multiple linear regression to assess the impact of the two adjustment variables, i) delay between last immunization and blood sampling and ii) age at blood sampling, on the humoral response described. This analysis highlights that these variables could not solely explain the lack of significant difference observed in the humoral response between individuals with a Delta-BA.1 co-infection and those with a BA.1 infection or the significant difference observed between both of these groups with the COVID-19-naïve fully vaccinated group.

	**Anti-RBD IgG levels (BAU/mL)**	**Anti-S1 IgA (µg/mL)**	**$PRNT_{50}$ -19A**	**$PRNT_{50}$-Delta**	**$PRNT_{50}$-BA.1**	**$PRNT_{50}$-BA.4**	**$PRNT_{50}$-BA.5**
**Delay between last immunization[1] and blood sampling for humoral investigation**	p-value= 0.4159	p-value= 0.4459	p-value=0.0412	p-value=0.9511	p-value=0.7228	p-value=0.3340	p-value=0.8880
**Age at blood sampling**	p-value=0.6509	p-value=0.8831	p-value=0.3221	p-value=0.9040	p-value=0.9775	p-value=0.9641	p-value=0.9028

[1] Last immunization corresponds to the infection for both groups with a breakthrough infection and the third vaccine injection for the COVID-19-naïve fully vaccinated group.

Supplementary Flow Chart: Flow chart detailing the inclusion process of the 9 Delta-BA.1 co-infected individuals.

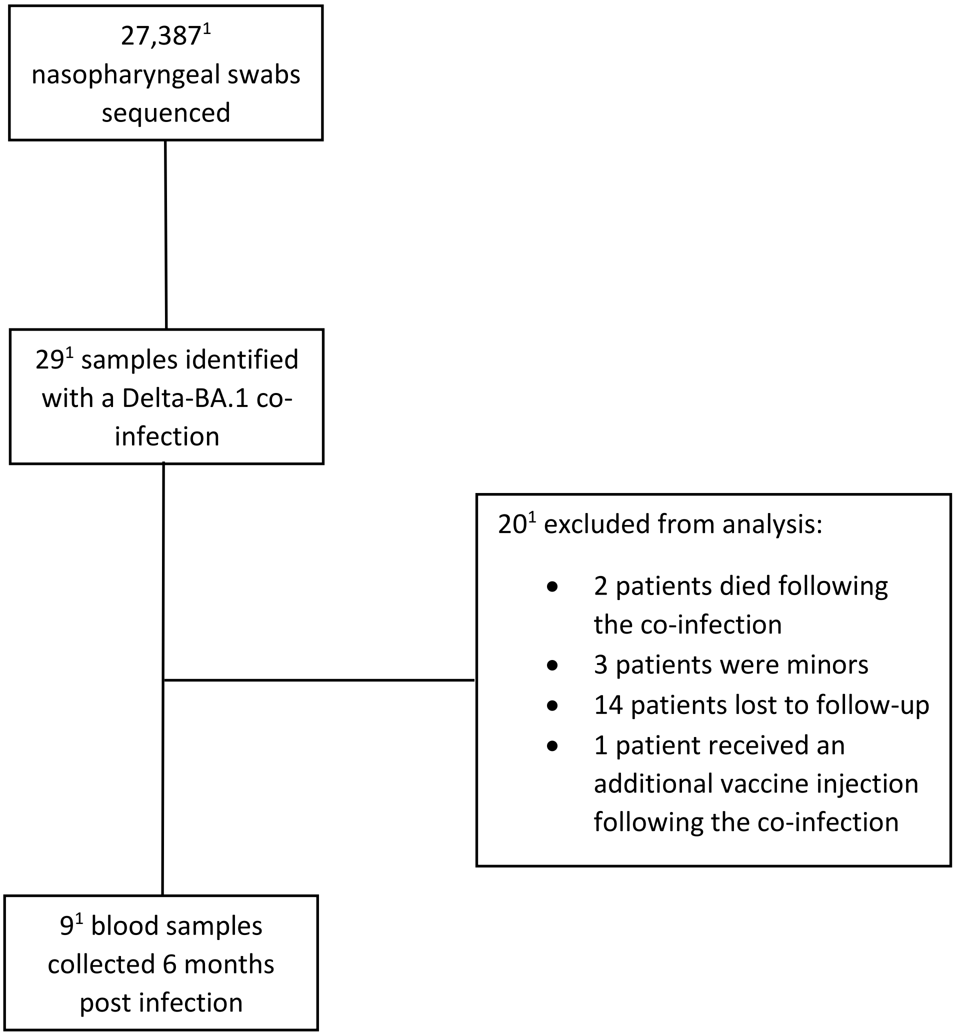

[1] Patients were first included in the *Bal A et al.,* Nat Commun, 2022[6], study where researchers identified 29 cases of Delta-BA.1 co-infections
