# Supplementary material for: Long-term humoral response following Delta and Omicron BA.1 co-infection
Source: NPJ Vaccines. 2023 Apr 20;8:57. doi: 10.1038/s41541-023-00652-8 (PMC10116110; doi:10.1038/s41541-023-00652-8)
Supplement: Supplementary file 2 — REPORTING SUMMARY [file 41541_2023_652_MOESM2_ESM.pdf]

nature portfolio

Corresponding author(s): Sophie Trouillet-Assanr
Last updated by author(s): Feb 15, 2023

Reporting Summary

Nature Portfolio wishes to improve the reproducibility of the work that we publish. This form provides structure for consistency and transparency in reporting. For further information on Nature Portfolio policies, see our Editorial Policies and the Editorial Policy Checklist.

Statistics

For all statistical analyses, confirm that the following items are present in the figure legend, table legend, main text, or Methods section.

n/a	Confirmed	
☐	☒	The exact sample size (*n*) for each experimental group/condition, given as a discrete number and unit of measurement
☐	☒	A statement on whether measurements were taken from distinct samples or whether the same sample was measured repeatedly
☐	☒	The statistical test(s) used AND whether they are one- or two-sided<br>*Only common tests should be described solely by name; describe more complex techniques in the Methods section.*
☐	☒	A description of all covariates tested
☐	☒	A description of any assumptions or corrections, such as tests of normality and adjustment for multiple comparisons
☐	☒	A full description of the statistical parameters including central tendency (e.g. means) or other basic estimates (e.g. regression coefficient) AND variation (e.g. standard deviation) or associated estimates of uncertainty (e.g. confidence intervals)
☐	☒	For null hypothesis testing, the test statistic (e.g. *F*, *t*, *r*) with confidence intervals, effect sizes, degrees of freedom and *P* value noted<br>*Give P values as exact values whenever suitable.*
☒	☐	For Bayesian analysis, information on the choice of priors and Markov chain Monte Carlo settings
☒	☐	For hierarchical and complex designs, identification of the appropriate level for tests and full reporting of outcomes
☒	☐	Estimates of effect sizes (e.g. Cohen's *d*, Pearson's *r*), indicating how they were calculated

Our web collection on statistics for biologists contains articles on many of the points above.

Software and code

Policy information about availability of computer code

Data collection	Clinical data were recorded by a trained Clinical Research Associate using Clinsight software (version _ Csonline 7.5.720.1).
Data analysis	Statistical analyses were performed using the GraphPad Prism® software (version 8; GraphPad software, La Jolla, CA, USA) and R software, version 3.6.1 (R Foundation for Statistical Computing, Vienna, Austria).

For manuscripts utilizing custom algorithms or software that are central to the research but not yet described in published literature, software must be made available to editors and reviewers. We strongly encourage code deposition in a community repository (e.g. GitHub). See the Nature Portfolio guidelines for submitting code & software for further information.

Data

Policy information about availability of data

All manuscripts must include a data availability statement. This statement should provide the following information, where applicable:

- Accession codes, unique identifiers, or web links for publicly available datasets
- A description of any restrictions on data availability
- For clinical datasets or third party data, please ensure that the statement adheres to our policy

The datasets generated and/or analyzed during the current study are available from the corresponding author on reasonable request. GISAID accession numbers for the 19A, Delta, BA.1, BA.4 and BA.5 strains used are EPI_ISL_1707038, EPI_ISL_1904989, EPI_ISL_7608613, EPI_ISL_12396843, and EPI_ISL_12852091, respectively.

Human research participants

Policy information about studies involving human research participants and Sex and Gender in Research.

Reporting on sex and gender	Sex information of participants has been collected and presented in supplementary table S2
Population characteristics	Population characteristics such as sex, age distribution and comorbidities of the study participants are presented in table S2.
Recruitment	Participants were included 6 months following the diagnosis with a breakthrough infection caused by either Delta and BA.1 simultaneoulsy or BA.1 only and after informed consent was obtained. As for vaccinated individuals, they were included 6 months after their third vaccine injection and after informed consent was obtained.
Ethics oversight	Approval was obtained from the regional review board in April 2020 (Comité de Protection des Personnes Sud Méditerranée I, Marseille, France; ID-RCB 2020-A00932-37; ID-RCB 2021-A01877-34).

Note that full information on the approval of the study protocol must also be provided in the manuscript.

Field-specific reporting

Please select the one below that is the best fit for your research. If you are not sure, read the appropriate sections before making your selection.

☒ Life sciences ☐ Behavioural & social sciences ☐ Ecological, evolutionary & environmental sciences

For a reference copy of the document with all sections, see nature.com/documents/nr-reporting-summary-flat.pdf

Life sciences study design

All studies must disclose on these points even when the disclosure is negative.

Sample size	We did not perform sample size calculations as we were only able to include 9 individuals co-infected with Delta and BA.1 due to the rarity of such infections. We included 9 individuals infected with only BA.1 and 9 vaccinated with three doses of the BNT162b2 vaccine in order to be homogeneous among the three groups
Data exclusions	No data were excluded from the analyses
Replication	For the measurment of anti-RBD IgG levels, samples were tested as simplicate since we used validated commercial ELFA kits and internal quality controls ensure reproducibility. As for the anti-S1 IgA measurement assay and the neutralizing antibody assay, samples were tested in duplicates
Randomization	They were allocated in each group on the basis of their immunization scheme (ie Delta-BA.1 co-infection vs BA.1 infection vs COVID-19-naive with three vaccine injections).<br>For immunological assays, samples were blinded and randomized.
Blinding	Coded samples were used for all experiments thus investigators were blinded to group allocation.

Reporting for specific materials, systems and methods

We require information from authors about some types of materials, experimental systems and methods used in many studies. Here, indicate whether each material, system or method listed is relevant to your study. If you are not sure if a list item applies to your research, read the appropriate section before selecting a response.

Materials & experimental systems

n/a	Involved in the study	
☐	☒	Antibodies
☐	☒	Eukaryotic cell lines
☒	☐	Palaeontology and archaeology
☒	☐	Animals and other organisms
☐	☒	Clinical data
☒	☐	Dual use research of concern

Methods

n/a	Involved in the study	
☒	☐	ChIP-seq
☒	☐	Flow cytometry
☒	☐	MRI-based neuroimaging

Antibodies

Antibodies used	Elisas<br>Recombinant protein Spike S1-His: SARS-CoV-2 (2019-nCoV) -Cat: 40591-V08H (Sino-biologicals) coating 1 microgr/ml<br>Goat Anti-human IgA (alpha chain specific)-HRP (SIGMA)--Cat: A0295-1ML dilution 1/10000
Validation	All antibodies used in this study are commercially available, and all have been validated and quality checked by the manufacturers and used in other published works

Eukaryotic cell lines

Policy information about cell lines and Sex and Gender in Research

Cell line source(s)	Vero E6 cells (ATCC CRL-1586)
Authentication	We did not authenticate the cell lines, but such authentication is performed by the supplier (ATCC) as part of the quality control
Mycoplasma contamination	All cell lines were regularly screened and tested negative for mycoplasma using a commercial kit (Lonza MycoAlert kit) # LT07-418
Commonly misidentified lines (See ICLAC register)	no commonly misidentified cell lines were used in the study

Clinical data

Policy information about clinical studies

All manuscripts should comply with the ICMJE guidelines for publication of clinical research and a completed CONSORT checklist must be included with all submissions.

Clinical trial registration	NCT05060939, NCT04341142
Study protocol	Study protocol is available upon request
Data collection	Participants with a breakthrough infection caused by Delta-BA.1 simultaneously or BA.1 only blood sampling was perfomed between june and august 2022. As for individuals who are COVID-19 naive and triple vaccinated, blood sampling was perfomed between march and may 2022.
Outcomes	The primary outcome was the positivity of the SARS-Cov-2 serological test and quantification of anti-RBD IgG and anti-S1 IgA, and the secondary outcome was the serum neutralization capacity.
